# Amyloid-β breaks brain's neuroprotection

**DOI:** 10.18632/oncotarget.18246

**Published:** 2017-03-28

**Authors:** Yunling Wang, Xike Qin, Hemant K. Paudel

**Affiliations:** Lady Davis Institute for Medical Research, Jewish General Hospital, McGill university, Montreal, Quebec, Canada

**Keywords:** Alzheimer's disease, amyloid-b, clusterin, sortilin, lysosome

Alzheimer's disease (AD) is the major cause of dementia in elderly population. Neuropathological features of AD are the extracellular plaques and intracellular neurofibrillary tangles (NFTs). Plaques are composed of amyloid-β (Aβ) peptide and NFTs contain hyperphosphorylated tau protein [1]. Genetic and biochemical studies suggest that extracellular Aβ deposition triggers pathological events leading to neurodegeneration and perhaps dementia [2]. Many pathological events downstream of Aβ however are still not clear.

Genome-wide association studies have identified clusterin (also known as Apo-J) as one of the major risk genes for late onset type of AD (LOAD) that represents ∼95% of all AD cases [2]. Clusterin is a member of heat shock family. The clusterin gene is highly conserved and is widely expressed in many organs and tissues where it participates in physiological processes including cell cycle, inflammation, lipid transport, membrane recycling, apoptosis and cell adhesion [3]. More importantly, clusterin binds to Aβ and prevents Aβ aggregation. In addition, clusterin enhances lysosomal Aβ degradation and promotes Aβ clearance through the blood brain barrier [3]. Aβ is also produced in normal brain at low level. One of the mechanisms by which brain keeps Aβ level checked is by maintaining relatively high level of clusterin [3]. In AD brain however, Aβ progressively accumulates and causes neurodegeneration [2].

In a recent study published in Neurobiology of Aging we showed that in hippocampal primary neurons, Aβ promotes lysosomal degradation of clusterin [4]. Aβ first induces the expression of lysosomal sorting protein sortilin. Sortilin subsequently, binds to clusterin and delivers clusterin to lysosome for degradation. Our study has revealed a novel Aβ-induced pathological cascade involved in the breakdown of a neuroprotective mechanism of the brain.

Clusterin is mainly a secretory protein coded by a single copy gene which is co-translationally transported from lumen of endoplasmic reticulumn where after glycoslation and cleaveage at Golgi, a heterodimer of α/β subunits connected by disulfide bridges is secreted. In addition, clusterin is also found in the cytosol in unglycosylated form. Secreted clusterin by binding to LRP2/ megalin receptor is internalized and subsequently targeted to lysosome for degradation [5]. Cytosolic clusterin on the other hand is targeted for degradation directly from Golgi apparatus [6]. Sortilin is located at both Golgi apparatus and plasma membrane. Future studies will be required to determine how sortilin trafficks secreted and cytosolic cluterin to lysosome.

Studies have revealed that clusterin is also present in the nucleus. Previously, the nuclear clusterin was shown to be formed by alternate splicing of clusterin mRNA. However, recent studies have shown that under certain stress conditions, full-length clusterin evades secretary pathway and accumulates in the cytosol [6]. When cellular calcium is depleted, cytosolic clusterin is cleaved, and translocates to nucleus and promotes apoptosis [7]. Aβ peptide induces stress, causes calcium depletion and induces apoptosis in neurons. It will be interesting to determine if Aβ promotes translocation of cytosolic clutsrein to the nucleus and if nuclear clusterin plays any role in Aβ-induced apoptosis in neurons.

Finally, tau is hyperphosphorylated in AD brain. Tau hyperphosphorylation causes microtubule instability, NFT formation an loss of axonal transport [1]. Cytosolic clusterin binds to tau and this binding is increased in AD brain [8]. Further studies will be required to determine if cytosolic clusterin has any role in promoting tau phosphorylation and/ or tau fibrillization in AD.
